# Community participation during two mass anti-malarial administrations in Cambodia: lessons from a joint workshop

**DOI:** 10.1186/s12936-018-2202-z

**Published:** 2018-01-27

**Authors:** Thomas J. Peto, Mark Debackere, William Etienne, Lieven Vernaeve, Rupam Tripura, Gregoire Falq, Chan Davoeung, Chea Nguon, Huy Rekol, Lorenz von Seidlein, Arjen M. Dondorp, Nou Sanann, Phaik Yeong Cheah, Martin De Smet, Christopher Pell, Jean-Marie Kindermans

**Affiliations:** 1 Mahidol-Oxford University Tropical Medicine Research Unit, Faculty of Tropical Medicine, Mahidol University, Bangkok, Thailand; 2 Centre for Tropical Medicine and Global Health, Nuffield Department of Medicine, University of Oxford, Oxford, UK; 3 Médecins Sans Frontières, Brussels, Belgium; 4 Center of Tropical Medicine and Travel Medicine, Department of Infectious Diseases, Academic Medical Center, University of Amsterdam, Amsterdam, The Netherlands; 5 Provincial Health Department, Battambang, Cambodia; 6 Centre for Parasitology, Entomology and Malaria Control, Phnom Penh, Cambodia; 7 Centre for Social Science and Global Health, University of Amsterdam, Amsterdam, The Netherlands; 8 Amsterdam Institute for Global Health and Development, Amsterdam, The Netherlands

**Keywords:** Cambodia, Community engagement, Dihydroartemisinin–piperaquine, Malaria elimination, Mass drug administration, *Plasmodium falciparum*

## Abstract

Two mass drug administrations (MDA) against falciparum malaria were conducted in 2015–16, one as operational research in northern Cambodia, and the other as a clinical trial in western Cambodia. During an April 2017 workshop in Phnom Penh the field teams from Médecins Sans Frontières and the Mahidol-Oxford Tropical Medicine Research Unit discussed lessons for future MDAs.

## Background

The emergence of artemisinin-resistant *Plasmodium falciparum* in the Greater Mekong Sub-Region (GMS) has prompted the malaria community to consider alternative strategies to accelerate falciparum malaria elimination [1]. Mass drug administrations (MDA) have been proposed to rapidly eliminate *P. falciparum* infections from transmission foci [2]. MDA entails offering an artemisinin-based combination therapy (ACT) to all members of a target population, regardless of malaria infection status, with the aim of interrupting local transmission. The intervention does not rely on diagnostic tests, which require time to complete, skilled labour to process and add costs. The success of this strategy depends on characteristics of the anti-malarial regimen, local transmission dynamics and uptake in target communities [3].

Achieving sufficiently high population coverage to interrupt *P. falciparum* transmission is challenging for several reasons. First, delivering a multi-day drug regimen to all members of a community requires considerable human resources. Second, potential target communities, where malaria transmission persists in the GMS, are often isolated, poor and mobile: characteristics that compound the logistical challenges. Third, in these areas, cases of clinical *P. falciparum* have often decreased in recent years, and populations might question the benefits of taking an anti-malarial when seemingly healthy.

Between 2015 and 2016, the Cambodia National Malaria Control Programme (CNM) participated in two pilot studies to evaluate the impact of mass administration of dihydroartemisinin/piperaquine (DHA–PQP) on local *P. falciparum* transmission. In 2015 and in partnership with Médecins Sans Frontières (MSF)—under implementation conditions—the acceptability, feasibility and impact of MDA on clinical malaria and Rapid Diagnostic Test (RDT)-positivity among febrile patients was assessed in Preah Vihear Province, northern Cambodia. In 2015 and 2016, a Mahidol-Oxford Tropical Medicine Research Unit (MORU)-led clinical trial in Battambang Province, western Cambodia, assessed the impact of MDA on clinical malaria cases, and used repeated cross-sectional surveys to monitor asymptomatic malaria infections. Levels of uptake in the target communities differed between the two sites: 72–93% of the *de jure* adult residents participated in the MDA in Battambang [4], compared to between 4 and 76% in Preah Vihear of the de facto population.

The contrasting levels of participation offer an opportunity to analyse how particular features of these MDAs, including the accompanying community engagement activities, and the local social context can influence uptake. During a joint workshop in April 2017, drawing on the ancillary social science research, members of the implementing teams collaboratively analysed the community responses to MDA at both sites. This article presents the results of this workshop.

## Settings

### Battambang

In Battambang Province, the MDA was conducted in Samlout District, part of an area targeted for the elimination of multi-drug-resistant *P. falciparum* [5]. With a decline in clinical *falciparum* malaria over the past 15 years, malaria transmission in Battambang is currently unstable [6]. In this border region, clinical and asymptomatic *P. falciparum* infections are associated with travel to local forests and a large proportion of transmission may occur outside villages [7, 8]. In Samlout District a programme of activities aimed at containing artemisinin-resistance was already ongoing including the recruitment and training of village malaria workers (VMWs); the distribution of effective anti-malarials, rapid diagnostic tests, long-lasting insecticide-treated bed nets (LLINs) and hammock nets to stable and mobile/migrant populations; and engagement with private drug sellers to combat counterfeit and substandard anti-malarials and to enforce the ban on the sale of anti-malarial monotherapies [5].

The study site comprised four villages selected on the basis of prevalence of asymptomatic *P. falciparum* infections detected in earlier prevalence surveys, their size, accessibility and recent clinical *P. falciparum* cases detected by village malaria workers in 2013–14 [9]. The selected villages recorded populations of between 239 and 1112 residents and total incidence of clinical malaria (all species) between 27 and 109 cases per 10,000 (2015). The target population was 2366 people. Local activities by the MDA team included, prior to MDA, and then at 3-monthly intervals for 12 months, cross-sectional surveys with venous blood sampling to detect asymptomatic *Plasmodium* prevalence and record health status. During the MDA, MDA staff also undertook weekly supervision of village malaria workers, collection of treatment records and identification of patients.

### Preah Vihear

In Preah Vihear, MSF conducted MDA in Chey Saen District, Preah Vihear Province. This area experiences seasonal transmission with a peak at the end of the rainy season, around November. The area has seen increasing artemisinin resistance [10] and transmission levels have recently decreased to a very low level, which occurs mostly outside the villages [11]. Passive Case Detection was built up in 2014, with VMW meetings, village chief and commune chief meetings through the support and strengthening of the District network of 20 VMWs, plus three health centres, two health posts and seven private registered providers. Reactive Case Detection (RACD): household screening with RDTs around *P. falciparum* cases was set up at the same time. The MDA site comprised eight villages (with between 529 and 2024 inhabitants) selected on the basis of their prevalence of asymptomatic *P. falciparum* infections and the concomitant presence in each of the villages of artemisinin-resistant parasites (C580Y mutation in the K13 domain of *P. falciparum*) detected in earlier prevalence surveys. The target population was 7583 people. The aim was to explore whether it would be possible to scale up MDA (Table 1).

**Table 1:** Summary of MDA characteristics at both sites

	Battambang	Preah Vihear
Sites	Four villages (two intervention villages in 2015 and two intervention villages in 2016). Total population 2366	Eight intervention villages simultaneously Total population 7583
Site selection	Selected based on *P. falciparum* prevalence survey results and village malaria worker treatment records	Selected based on *P. falciparum* prevalence survey results and presence of K13 mutated parasites
Anti-malarial	DHA–PPQ	DHA–PPQ
Preparation period	Long (8 weeks, plus 4 weeks in the study villages): discussions on strategy and safety delayed the protocol approval and in effect increased preparation time	Short (about 6 weeks) Late approval of the protocol reduced the time available to plan the project, planned to be done in the dry season
Drug administration procedure	Central. DOT by local health centre nurses (central village location in 2015; at a central location or house-to-house in 2016)	House to house. DOT ensured by teams going from house to house
MDA schedule	Three doses over 3 days—at monthly intervals for 3 months. Post-MDA, weekly identification of newcomers, especially forest returnees. A single 3-day course of DHA–PPQ offered new arrivals 12 months following MDA	Three doses over 3 days—at monthly intervals for 3 months. Decision taken to halt after low participation, MDA stopped after round one
Timing of MDA	July to September (early rainy season) 2015 in the first two villages and 2016 in the remaining two villages	March–April 2015 (pre-rainy season)
Safety monitoring and follow-up	Direct solicitation of adverse events over 3 days of drug administration by local health centre nurses, and again on the 7th day by village volunteers to record and assist with any adverse events. 30 days of passive follow up	External medical teams (two MoH nurses/village—two MSF physician) present in villages during 3 days of DOT + 2 days later follow up adverse events. MSF nurses were also present 24 h per day in health facilities during intake 1-month post-MDA
Informed consent	Community meetings and census used to explain study to each household head. Individual written signed consent	Community meetings to explain MDA. Verbal consent from community leaders and household heads. Written consent from individual participants
Incentives	KHR 10000 (~ US$2.5) per round and participant for round 1 (none for rounds 2 and 3 in 2016)	None
Other benefits	Free health service for minor conditions during MDA, provided by local health centre staff and supervised by a study physician	Healthcare was provided by an MSF nurse and MD in health facilities (additional to usual local staff). Transport was provided to referral facilities. Other medical costs were reimbursed until 1 month after intake

*DHA–PPQ* dihydroartemisinin–piperaquine, *DOT* directly observed treatment, *MDA* mass drug administration, *MoH* Ministry of Health, *PCD* passive case detection, *MD* medical doctor, *MSF* Medecins sans frontiers

## Mass drug administration

At both sites, staff evaluated the feasibility and effectiveness of treating entire villages with DHA–PQP, at the time, the first-line anti-malarial. In Battambang, three rounds of ACTs over 3 days were administered in monthly intervals under direct observation. The MDA was conducted as a clinical trial, with randomization at village level. In 2015, MDA was conducted in two intervention villages and in 2016 the control villages received MDA. In Preah Vihear, MDA used the same regimen. The intervention was halted after the first round because of the low coverage in the target villages.

## Community engagement

At both sites, a range of community engagement activities accompanied the MDAs. Their aims were to communicate with communities about the malaria, the aims of MDA, and to promote coverage in the target villages. Table 2 provides a summary of these activities.

**Table 2:** Community engagement activities according to target groups

Target groups	Activities	
	Battambang	Preah Vihear
National/regional authorities	Involvement of national malaria control programme and provincial health authorities in implementation and supervision. Collaboration with district and commune authorities including individual leaders already known to study communities	Support letters were obtained from National Malaria programme. Provincial Health Department and District Governor were informed. Verbal support of commune chiefs and village chiefs was received during village leaders meetings
Volunteers/guides	Teams consisting of village leaders, malaria workers, and volunteers implemented MDA in each village. Volunteers supervised a block of ~ 10–30 neighbouring houses. Volunteers assisted with contacting households, providing information and invitations, helping with MDA, post-MDA follow-up and identifying newcomers	Local volunteers were recruited and trained from communities to inform villagers and to guide the social workers house-to-house. MSF trained social workers, mostly local students, in each village with assistance of village leaders/local guides
Village malaria workers	Provide diagnosis and treatment to clinical malaria cases (RDT on febrile participants) during MDA and surveys, supervise volunteers during follow-up, collaborate with medical team, alert study team and leaders to rumours/perceived adverse reactions of MDA and help sensitize participants	Provide diagnosis and treatment to clinical malaria cases, supported and guided by the passive case detection (PCD) team
Locally influential people	Small group and individual meetings with local political leaders, police, teachers, shop-keepers, private sector health care providers, traditional healers and military staff stationed in the area Monks were asked to bless activities and talk to their communities about working together and the importance of health	Public health department (PHD) director, District Governor, Commune Chiefs and village chiefs in the District were visited by the project coordinator and liaison officer. Health promotion officers met with village chiefs gained verbal consent. Identification of block leaders, to organize meetings with small groups of villagers
Whole community	Public engagement event with music, quiz, prizes, invited speakers, household gift packs and snacks (main mobilisation event prior to MDA 2015); Video performance, drama workshops, singing competition, public drama performance (main mobilisation event prior to MDA 2016)	Malaria movie and presentation MSF activities in all (sub) villages (Nov 2014); door-to-door visits by guides; block meetings; mass village meetings; leaflet distribution with key information about MDA
Forest goers	Small meetings, visits to forest, build trust, health education about forest-acquired malaria, contact in advance to avoid missing people	No specific actions taken
Women/mothers	Health education given in small groups, listen to specific fears about women or children taking medicine. Explain drug safety and adverse reactions and reasons for exclusion of pregnant and lactating mothers	No specific actions taken
School activities	Outdoor games, colouring-in competitions, and prizes to generate good feeling, raise awareness, and avoid children being scared of MDA	No specific actions taken
Non-participants	Followed up by study staff to determine if they could be persuaded to join, and if they were present or away from the village	Follow-up among individuals who had been absent, and individuals who stated they would not be present for the full DOT

*MDA* mass drug administration, *DOT* directly observed therapy

## Ancillary social science

In Battambang, qualitative data were collected alongside the 2015 MDA and the findings are described in detail elsewhere [12]. Additional qualitative data were collected immediately after the 2016 MDA to explore how changes in implementation affected coverage. A questionnaire-based survey was also undertaken in Battambang in 2015 and 2016 to identify factors associated with participation in the MDA. Following the MDA in Preah Vihear, a qualitative study was undertaken to explore why coverage had declined rapidly as the MDA progressed. This included meetings with all formal and informal leaders, after meetings in villages (smaller groups) and after again feedback to all leaders per village—in collaboration with the health department staff (deputy director, nurses, health centre chiefs, and hospital director). Table 3 gives an overview of the data collection techniques employed and respondent types.

**Table 3:** Ancillary social science data collection and respondents

	Battambang		Preah Vihear
	2015	2016	2015
Methods	Semi-structured interviews (40), focus group discussions (4), observations, exit poll (30); questionnaires (123)	Semi-structured interviews (28), focus group discussions (4), questionnaires (~ 240)	Semi-structured interviews (16), focus group discussions (13); Total 113 villagers meetings with community/community leader (16)
Respondents	MDA participants, village leaders, study staff, non-participants/drop-outs, forest-workers	Study participants, village leaders, study staff, non-participants/study drop-outs	MDA participants, non-participants, village leaders, household heads, MDA staff (social workers, PHD nurses)

*In Battambang*, respondents for the ancillary social science study were selected based on a random sample of households in the de facto population (MDA participants), using a directed (forest-goers who were selected with the assistance of village leaders) and exhaustive approach (village leaders and study staff). Study records were used to identify villagers who did not participate or dropped-out from the study, and a random sample was selected and interviewed.

*In Preah Vihear*, respondents were purposively selected from four intervention villages. The recruitment strategy aimed to identify participants and non-participants, adolescents, adults and elders of both genders. Villages were selected by level of participation (high and low), and high number of medical consultations and calls to the medical team after the intervention.

Interviews and focus group discussions were audio recorded and transcribed verbatim and translated into English. For the data from the two sites, qualitative content analysis was undertaken separately using qualitative data analysis software NVivo 10 Software (QSR International Pty Ltd). The analysis process used analytical categories based on initial research questions and substantive issues that emerged during line-by-line coding with a pre-developed codebook. The outcome of this process was then discussed, and the findings compared and contrasted at the joint workshop held in April 2017.

## Findings from the ancillary social science research

At both sites, staff had undertaken activities prior to the MDA: malaria prevalence surveys (which entailed blood draws) and, in Preah Vihear, nurses had been present in the villages for over a year (training, supervising treatment and RACD). At both sites, the previous activities were positively evaluated by villagers and village leaders.

### Perceptions of the MDA and implementing organization

At both sites, villagers were familiar with the aim of the MDA “to eliminate” malaria. They were however often less aware of the intervention rationale—to treat asymptomatic infections. During the community engagement was the first time that most community members had heard messages about ‘drug resistant malaria’ and ‘asymptomatic carriers’. In Battambang, some members of the field team were confused about the science that underpinned the intervention. At both sites, previous treatment guidelines that emphasised diagnosis of malaria before giving anti-malarials prompted some confusion.

During the MDA, MORU became a well-known term. Community members also described generally positive opinions of staff’s efforts at eliminating malaria. Nonetheless, staff interpreted the drop in coverage during round 2 in 2015 as an indication that attitudes towards MORU had changed and that they had begun to see the team as outsiders. In response, representatives from the local health authorities took up more prominent roles in the third round of MDA.

In Preah Vihear, before the intervention, the initial description and notion of MDA was well-received by members of the target community, though it was viewed as a last-minute introduction. In addition to MDA prompting confusion regarding previous messages about overtreatment, MSF staff gained the impression that the non-governmental organization (NGO) was primarily associated with the treatment of symptomatic malaria. Few respondents made the link between MDA and the survey conducted the year before. Although the organization was well known, and their efforts to eliminate malaria positively received, it is possible that the change in approach and the new activities raised questions among villagers.

### Financial incentives

Incentives were provided in cash for participants in the Battambang MDA in all three rounds in 2015 and the first round in 2016. Intended to compensate participate for a day’s labour, the incentives were however not readily cited by villagers as a major reason for participation. The absence of financial incentives for two rounds of MDA in 2016 did not negatively impact coverage. No financial incentives were provided in Preah Vihear.

### Written informed consent

Amongst villagers, the process of informed consent for MDA was sometimes viewed with suspicion and this raised concerns about the MDA. In Preah Vihear, a few villagers misunderstood the Khmer terms used for surveys and MDA, interpreting it as having connotations of experimentation and that participants would ‘discharge liability’. Learning from the experience of staff working in Preah Vihear, in the Battambang MDA, a more neutral term, “project” was used. Both MDAs required signed consent. In Preah Vihear, for a few villagers, this stoked fears that the signature could be used for political reasons or land grabbing. Such fears were not mentioned in Battambang.

The information sheet that accompanied the informed consent in Preah Vihear provided extensive details of the potential (albeit unlikely) adverse reactions of the anti-malarials. Staff suggested that this contributed to the range of health complaints that villagers reported after MDA (see below). Staff in Battambang were informed of this and adapted their information sheet accordingly to reflect the low likelihood of adverse reactions.

### Perceived adverse reactions

In Battambang, MDA was conducted during the rainy season and coincided with seasonal bouts of mild cold and influenza-like illnesses. Some villagers attributed these illnesses to the anti-malarial and this had an impact on coverage in the second round, with an estimated 10% decrease in participation. Although study staff viewed the decline as a challenge, they were able to give an understandable explanation for the minor health complaints that villagers were experiencing at the time of MDA.

In Preah Vihear, MDA was conducted towards the end of the dry season and staff had no such easy explanation for the complaints that villagers experienced at the time of the MDA. In fact, staff, particularly the locally recruited village guides struggled to respond to villagers’ questions about perceived adverse reactions. News of adverse events spread through the villages and staff described them as a major reason why coverage declined in target villages. Based on history and physical examinations, MDA health staff confirmed that almost all of these complaints were unlikely to be related to the MDA.

At both sites, concerns about the adverse events that villagers attributed to the anti-malarial were compounded by several factors. First, many adverse events were seen as requiring medical attention—and in some cases in Battambang, because they did not seek help from the MDA health staff, this was viewed as an additional cost. Second, the adverse events were seen as potentially limiting one’s capability to work and to earn needed income.

In Preah Vihear, the adverse events prompted changes in attitudes towards the MDA. These were linked to rumours suggesting that the anti-malarial was different from that used by the VMWs. Linked to the misunderstanding about the nature of the MDA as experimentation, some villagers viewed it is a dangerous and potentially targeted at the Khmer people. Private practitioners were described as participating in spreading these stories.

The number of villagers who sought assistance at health facilities in Preah Vihear was so large that they were unable to deal with all requests for treatment. This may have been related to the presence of extra MDA nurses and an expatriate physician. Staff explained that participants could report and seek assistance for any health problem until 1 month after the last MDA dose and some villagers associated all symptoms/medical complaints during this period with the MDA. The availability of free healthcare and the presence of an ambulance and expatriate physician may have heightened concerns about the anti-malarial. There were also complaints about the treatment that was offered to palliate adverse events and about the costs of travelling to health facilities for this assistance, even those were reimbursed in both sites at a later stage.

In Battambang, people were concerned about having to purchase intravenous (IV) drug “kits” from private health providers to manage perceived adverse reactions to the study drugs. Although expensive, these kits were popular because of their perceived “powerful energising” effects and because they could be administered by a friend or neighbour (former soldiers who had received some medical training).

At both sites, villagers were also worried about the study because of the widely reported 2014 case of negligent needle practice by a health provider in Battambang Province, which led to multiple HIV infections [13]. Staff in Battambang described this as a challenge during community engagement but it was not described as a reason for non-participation amongst villagers. In Preah Vihear, however, it was mentioned by villagers as a reason not to participate.

### Community engagement

Local community engagement activities began around 6 weeks before the start of the MDA in Battambang and 3–4 weeks before in Preah Vihear. For the Battambang team, delays in the ethical approval procedure gave additional time for planning the community engagement (and the opportunity to incorporated lessons from the Preah Vihear). In Preah Vihear, staff—and villagers—were critical of the lack of time available during the community engagement prior to the MDA to provide adequate information. Field staff in Battambang also described being rushed to provide adequate community engagement.

Differences in the number of personnel who were responsible for community engagement explain part of this: in Battambang a team of five (later six) field staff were engaged specifically in the community engagement, and senior members of the study staff also took active roles in community engagement activities in the four villages; in Preah Vihear, two health promotion officers (daily workers, not fixed MSF staff, but trained for this specific task) were charged with delivering community engagement across eight villages (nurses were present in villages only during 3 days). The number of intervention villages also played a role.

Staff involved in the Battambang MDA adapted the community engagement to the local context, according to formative research prior to and during the MDA. This include initial interviews with local community leaders and semi-structured interviews with villagers 1 week after each round of MDA. The semi-independent staff who conducted interviews were able to feedback information to the team who implemented MDA. The presence of the study team in the villagers during 7 days after each round of MDA meant that they were able to respond to complaints and concerns in small gatherings or house-to-house visits.

This capacity to adapt community engagement in Battambang led staff to alter their approach after the decline in participation in round 2 during the 2015 MDA. Prior to round 3, MORU staff took less prominent roles in meetings and rather local health staff from the government-run facilities led the events. Coverage subsequently increased in round 3 and this model based on local health staff was used in the 2016 MDA, during which coverage remained high (despite the removal of incentives in rounds 2 and 3).

In Preah Vihear social science research was conducted retrospectively to understand the reasons for the rapid decrease in participation in the MDA. MDA health staff were present in local facilities during and after the first round of MDA who were able to listen and respond to villagers’ concerns. Because the MDA was halted, community engagement was not adapted. The MSF team had initially assumed that gaining the approval of the village leaders, would be sufficient to ensure that all villagers would agree to participate. The leadership dynamics in each village were however more complex. Other formal and informal leaders (such as teachers, policemen, monks, and representatives of political parties) also influenced other people’s decisions about whether to participate in MDA.

### Malaria as a health concern

In Battambang, in spite of the recent declines in clinical cases, and a limited understanding of the disease, malaria was a concern to villagers. This concern was often based on past experiences, for example, malaria was a major cause of ill health when some of the study villages were settled over 20 years ago. People also recognized that a bout of malaria has financial implications, particularly in terms of opportunity costs when a sick person is unable to work. Villagers were therefore generally receptive to malaria prevention interventions and appreciated the effort to ease the burden of disease in their communities.

In Preah Vihear, some villagers also described how they wished to avoid illness because of its financial implications. However, accustomed to the “test and treat” approach to malaria control, most people were more comfortable treating themselves when they were sick than taking an anti-malarial when feeling healthy.

## Discussion

Mass drug administration for the interruption of local malaria transmission can be challenging to implement for a number of reasons including the choice of anti-malarial drugs, the epidemiology of malaria, and the participation of the target communities. The experience of conducting MDA in villages in two regions of Cambodia highlights several important challenges in engaging the public so as to achieve a satisfactory coverage and provides potential signposts for future efforts to integrate this approach in a broader malaria elimination strategy.

### Learning from experience

The delayed start of the MDA in Battambang provided time for community engagement and for dialogue between the MDA teams. The team in Battambang had time to adapt elements of the study design and community engagement based on the experiences in Preah Vihear. Key revisions to the MDA procedures included: changing the term used to describe MDA from a word that could be interpreted as experimentation to a term more aligned with “project”; placing less emphasis on the potential adverse reactions of the anti-malarial used for MDA and no visible emergency health care interventions, such as an ambulance within the target villages. Staff at the Battambang site were also pre-warned of the potential impact of concerns about perceived adverse reactions. Hence, documenting the MDA process, analysing the challenges and communicating the lessons learned is key to maximizing the chances of success of this approach as part of future malaria elimination programmes.

### Communicating the risks of adverse reactions

At the time, the anti-malarial used for MDA, DHA–PQP, was the first-line treatment for uncomplicated falciparum malaria in Cambodia. This ACT, like all pharmaceuticals, has the potential to provoke adverse reactions, some of which are very rare but severe. In light of the “experimental” nature of MDA as an approach to malaria elimination, staff administering the ACT were obliged to outline the possibility of adverse reactions. In Preah Vihear, as a result of the requirements by the MSF ethical review board, MDA staff disclosed all possible adverse reactions and this contributed to unwarranted anxieties among villagers. In light of the MSF experience MDA staff in Battambang put less emphasis on the risk of adverse reactions during meetings. Adapted explanations combined with the reduced visibility of ancillary care in Battambang, may have left the villagers less concerned about taking the anti-malarial. Paradoxically, trying to reassure populations in Preah Vihear by installing a visible and easily accessible (available 24 h per day) medical presence may have created fears.

### Scale of MDA

Careful consideration for the size of the target population is needed when designing MDA. Because the overarching aim of the MDA was to look at the feasibility of scaling up, the target population was much larger in Preah Vihear: eight intervention villages with a total population of three times that of the four villages in Battambang (of which only half were targeted in MDA each year). This meant that concerns about adverse reactions and other aspects of the MDA that emerged in the early stage were difficult to manage: there were probably not enough staff members present for a long time in villages (they were in health centres) to ease adequately the worries and prevent them spreading to villages where MDA was subsequently conducted. Because staff in Battambang conducted MDA in two villages only, they were able to monitor and quickly respond to villagers’ concerns.

### Responsive (and inclusive) community engagement

Formative research enabled staff in Battambang to tailor the community engagement to the local communities—such research has also been highlighted as an important component of community engagement for mass anti-malarial administration in Laos [14]. Moreover, because they were present in target villages in sufficient numbers and accessible to villagers who could voice concerns staff were able to respond to specific worries about perceived adverse reactions. They were also able to adapt the overall community engagement strategy in response to a drop in coverage in round two; staff responded by asking representatives from the local health authority to take more prominent roles in village meetings and coverage in the subsequent round increased [12].

### Understanding community (health) concerns

A key element of the formative research is understanding how malaria is perceived in a target community. In Battambang members of target communities were worried about the direct (healthcare, transport) and indirect (opportunity) costs of a bout of malaria. They were therefore receptive to messages about the benefits of eliminating a troublesome illness from their villages. The research also provides the opportunity to gather data on how to adapt messages to local ideas about malaria, for example, what are the terms that resonate with people and how to pitch the benefit of the intervention. Such concerns were less pronounced in Preah Vihear: malaria incidence was low and the disease was not a pressing concern for the target population (even among those who were more at risk because they work in the forest).

### Incentives

The impact of financial incentives on uptake is equivocal. On the one hand, in Battambang, their absence did not have a negative impact during two rounds of MDA in 2016. However, complaints about perceived adverse reactions were compounded by the opportunity and direct costs e.g. missing a day of work and having to purchase an “IV kit”. The incentives are likely to mitigate at least the impact of these costs and might therefore reduce the vehemence with which people complain about the MDA.

### Local hierarchies and politics

In Preah Vihear, the team hoped that an approval of health authorities would ensure the participation of other community members based on the ubiquitous hierarchies in rural Cambodian life. However, villagers openly challenged the decisions of their leaders. Such questioning might also have been prompted by the position of the local opposition political party. In Myanmar, the fragmentation of a community along political lines also had implications for uptake of the MDA [15].

## Conclusions

The experiences of two teams conducting anti-malarial MDAs in two areas of Cambodia highlight the challenges and provide lessons how to employ this intervention as part of broader malaria elimination strategy. Subtle events such as the emergence of rumours can have a devastating effect on participation resulting in an insufficient coverage and preventing an impact on local malaria transmission. The impact of such subtle events is context specific—not all communities are as susceptible to rumours. The duration and quality of the preparation, the engagement of the target community is critical for success. Only if the majority community members are convinced that participating in the campaign is to their benefit—whether health, financial, social or political—can the people overcome their own concerns and the dissuasive messages of others. Investigating the dynamics of communities is critical for the implementation of health interventions such as MDA. The success of the programme depends on the ability to understand the local context—identify people’s concerns, monitor emerging rumours and examine the local political circumstances—and to respond appropriately.

## Abbreviations

ACT: artemisinin combination therapy
CNM: Centre for Parasitology, Entomology and Malaria Control, Cambodia
DHA–PQP: dihydroartemisinin–piperaquine
MDA: mass drug administration
MORU: Mahidol-Oxford University Tropical Medicine Research Unit
MSF: Médecins Sans Frontières
NGO: non-governmental organization
RACD: reactive case detection
RDT: rapid diagnostic test
VMW: village malaria worker
